# Bu-Shen-Zhu-Yun decoction induces PRLR deubiquitination and JAK2/STAT5 activation via CSN5 *in vitro*

**DOI:** 10.18632/aging.203426

**Published:** 2021-08-23

**Authors:** Hua Feng, Huifang Zhou, Jianxia Lu, Qing Zhang, Xingran Tang, Yujie Shang

**Affiliations:** 1 Institute of Rehabilitation, Jiangsu Vocational College of Medicine, Yancheng 224008, Jiangsu Province, China; 2 Department of Gynecology, Nanjing University of Chinese Medicine, Nanjing 224005, Jiangsu Province, China; 3 Department of Gynecology, Affiliated Hospital of Nanjing University of Chinese Medicine, Nanjing 210029, Jiangsu Province, China; 4 School of Clinical Medicine Jiangsu Vocational College of Medicine, Yancheng 224008, Jiangsu Province, China; 5 Nanjing University of Chinese Medicine, Nanjing 224005, Jiangsu Province, China

**Keywords:** Bu-Shen-Zhu-Yun decoction, hyperprolactinemia infertility

## Abstract

Purpose: To determine the effect of Bu-Shen-Zhu-Yun Decoction (BSZY-D) on the kisspeptin through JAK2/STAT5 signaling pathway in hyperprolactinemia (HPRL) infertility.

Method: SD rats were treated with BSZY-D for cerebrospinal fluid (CSF) extraction. GT1-7 cells were subjected to different treatments. The phosphorylation levels of JAK2 and STAT5, and the expressions of PRLR and kisspeptin of GT1-7 cells in different groups were detected by western blot, RT-qPCR and immunofluorescence. The expressions of CSN5 and GATA1 and other molecular features were checked by western blot, RT-PCR, co-immunoprecipitation and renilla luciferase activity.

Results: The phosphorylation levels of JAK2 and STAT5, and the expressions of PRLR and kisspeptin in the HPRL group were significantly decreased, and these changes could be reversed after BSZY-D treatment. In addition, the presence of PRLR deubiquitination was detected in the HPRL group, which could be reversed by shRNA-CSN5, suggesting that BSZY-D played a role through targeting CSN5. The binding level of GATA1 and CSN5 promoter in the HPRL group was significantly decreased, but elevated in the HPRL (BSZY-D/CSF) group (P < 0.05).

Conclusion: BSZY-D improved the transcription activity of GATA1 and increased the binding of GATA1 and CSN5. BSZY-D was involved in the deubiquitination of PRLR, which contributes to alleviating the symptoms of HPRL infertility.

## INTRODUCTION

Hyperprolactinemia (HPRL) is caused by a variety of factors and characterized by the significant elevation of serum prolactin (PRL). It is a syndrome of reproductive-endocrine disorder in the hypothalamic-pituitary axis, with relatively high morbidity [1]. At present, although there is no consistent detection method or reference range among different hospitals, it is widely accepted that the subject with serum PRL value more than 1.4 nmol/L should be diagnosed as HPRL [2]. The pathogenesis of HPRL includes the physiological, pathological, and pharmacological factors [3], among which the physiological factors include diet, sleep, gestation, nipple stimulation and breastfeeding; common pathological factors mainly include hypothalamic tumors, pituitary or ectopic prolactinoma, and acromegaly; as for pharmacological factors, some anesthetic (cocaine) and Chinese herbal medication (Angong Niuhuang Pill) can induce the HPRL. The main clinical symptoms include galactorrhea, menstrual disorders, hirsutism, and even infertility [4]. Nowadays, the medication such as bromocriptine is mostly applied in clinical practice. Although the application of bromocriptine has a certain curative effect, the side effects such as dizziness, nausea, vomiting, and resistance to dopamine receptor agonists cannot be ignored. Therefore, it is necessary to explore more safe and effective therapeutic strategies for patients with HPRL infertility.

The prescription of Bu-Shen-Zhu-Yun decoction (BSZY-D) created by Professor Guicheng Xia, is composed of yam, vinegar bupleurum, antlers tablets, red white peony root, cornus, dodder, salvia, angelica, amethyst, poria, fried cortex moutan, etc. This decoction has been proved to regulate menstruation and assist pregnancy [5]. BSZY-D has been widely applied in gynecological diseases, such as luteal phase defect, anovulatory infertility, and polycystic ovarian syndrome [5–7]. However, the efficacy of BSZY-D in HPRL infertility remains elusive.

Kisspeptin, a polypeptide hormone encoded by KISS-1 gene, can regulate the female reproductive endocrine functions. Kisspeptin modulates the secretion of gonadotropin-releasing hormone (GnRH) through the feedback effects of estradiol, and subsequently is involved in the regulation of reproductive and growth hormones in the reproductive system [8]. Hence, kisspeptin contributes to maintaining the stability of the hypothalamus-pituitary-ovarian (HPO) axis. A previous study has reported that BSZY-D can regulate the secretion of serum progesterone, follicle-stimulating hormone, luteinizing hormone, and estradiol through kisspeptin/GPR54 pathway, thereby improving luteal function and promoting embryo implantation [9].

PRL binds to its receptor PRLR and forms a complex that can further activate the Janus kinase 2 (JAK2). Subsequently, the activated JAK2 leads to its downstream signal transducer and activator of transcription 5 (STAT5) [10]. Phosphorylated STAT5 protein forms homodimers or heterodimerizes with other STAT family members, relating to the nucleus translocation and transactivation of the molecular target. JAK2/STAT5 signaling pathway is involved in the development of HPRL, and PRL/PRLR-JAK2/STAT5 signaling pathway is widely explored in cell proliferation, differentiation, and immune regulation. It can also initiate the transcription of certain target genes, and enable the expressions of ovarian and endometrial related receptors.

High PRL level elevates the combination of PRL and PRLR. On the one hand, it can activate the downstream JAK2/STAT5 pathway in a short time. On the other hand, it can lead to the ubiquitination, endocytosis, and degradation of PRLR, causing inhibition of PRL-mediated JAK2/STAT5 pathway in a long period. Excessive PRL cannot bind to its receptor, thus the expressions of JAK2/STAT5 pathway and its downstream kisspeptin are suppressed. CSN5, a type of deubiquitinating enzymes, can inhibit the degradation of PRLR through deubiquitination. We analyzed the transcription factors of CSN5 in the GENERADAR website (https://www.gcbi.com.cn/gcanalyze/html/generadar/index), and found that GATA1 is one of the transcription factors of CSN5. Hence, the improvement of the deubiquitination level of PRLR in patients with HPRL is the key for the treatment of infertility, miscarriage, irregular menstruation and so on. Therefore, the illustration of BSZY-D underlying mechanism in HPRL is the crucial part. However, no research has reported the relationship between PRLR deubiquitination and BSZY-D.

In the current study, we hypothesized that BSZY-D could improve the HPRL infertility through PRLR deubiquitination, targeting JAK2/STAT5 pathway and its downstream kisspeptin of hypothalamus. Our purpose is to explore the underlying molecular mechanism of HPRL infertility, thereby providing the new theoretical basis for the potential treatment application in clinic.

## RESULTS

### Effect of PRL on PRLR/JAK2/STAT5 pathway in GT1-7

**Figure 1:**
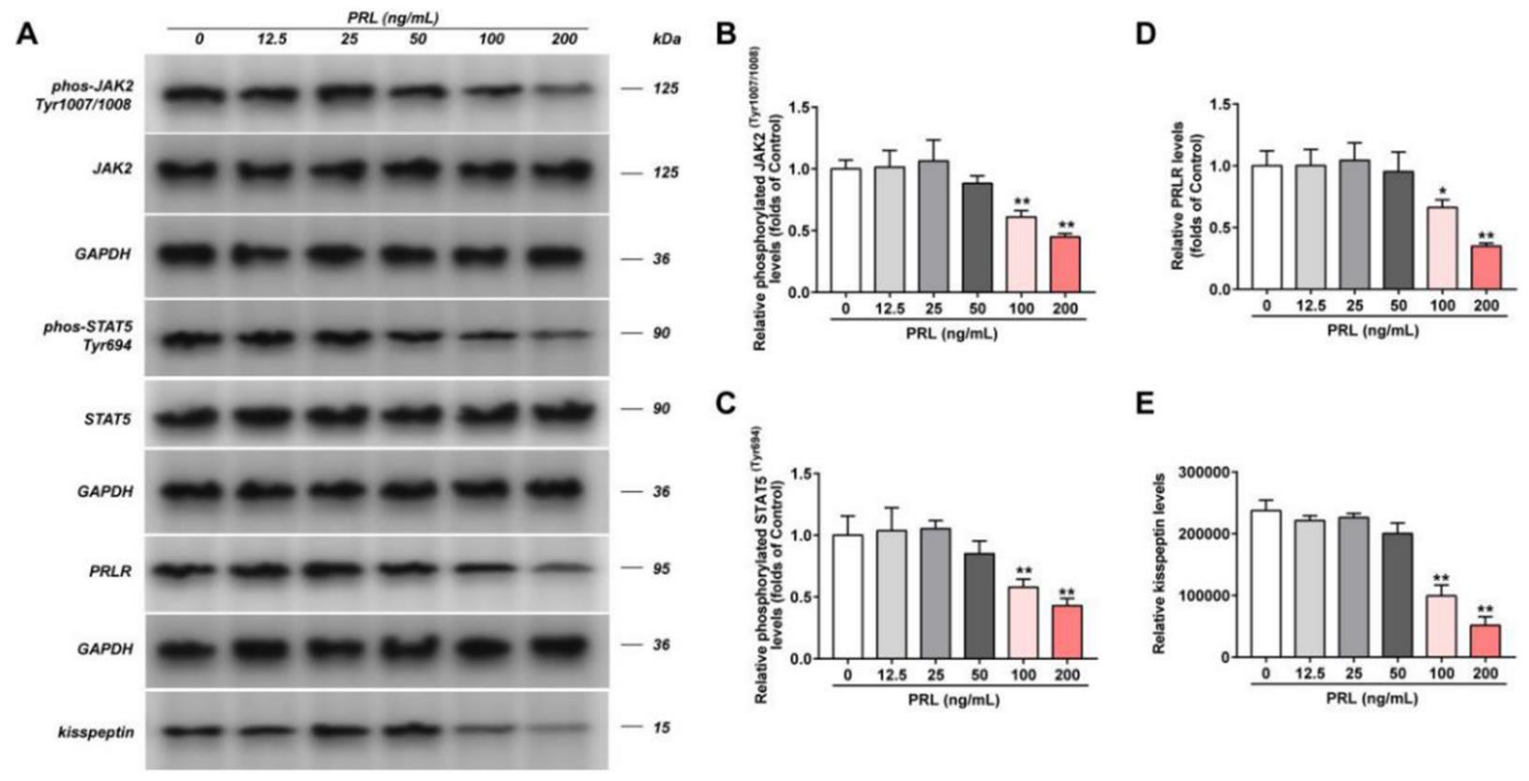
**Effect of PRL on PRLR-JAK-STAT5 pathway in GT1-7.** The phosphorylation levels of JAK, STAT5 and the levels of PRLR and kisspeptin in GT1-7 from different groups were detected by western blot assay, and representative bands were shown in (**A**). The phosphorylation levels of JAK (**B**) and STAT5 (**C**), and the level of PRLR (**D**), and the level of kisspeptin (**E**) were normalized to control. The results were presented as mean ± SD (n = 3). *p < 0.05, **p < 0.01 vs. Control group.

The viability of GT1-7 cells under different concentrations of PRL treatment was detected, and the results showed that PRL (400 ng/ml) has a significant inhibitory effect on the viability of GT1-7 cells, compared with the control group (P < 0.05). No effects have been found in any concentration of CFS on the viability of GT1-7 cells (Supplementary Figure 2). Hence, we used PRL at the concentration of 0 ng/mL, 12.5 ng/mL, 25 ng/mL, 50 ng/mL, 100 ng/mL, 200 ng/mL, and CFS at a concentration of 10%. Then the phosphorylation levels of JAK, STAT5, and the levels of PRLR and kisspeptin of GT1-7 cells in different groups were detected by western blot (Figure 1). Our data represented that compared with the Control group, the phosphorylation levels of JAK2 and STAT5, and the levels of PRLR and kisspeptin in the PRL (100 ng/ml and 200 ng/ml) group were significantly decreased (P < 0.05). Consistently, the mRNA expression of kisspeptin was significantly declined in the PRL (100 ng/ml and 200 ng/ml) group, but no apparent change was detected in PRLR mRNA expression (Supplementary Figure 3). Therefore, PRL was concentration-dependent. The higher PRL had a more significant effect, so we took 200 ng/mL PRL for the subsequent experiments.

### The regulation of BSZY-D on PRLR/JAK2/STAT5 pathway via CSF in GT1-7

**Figure 2:**
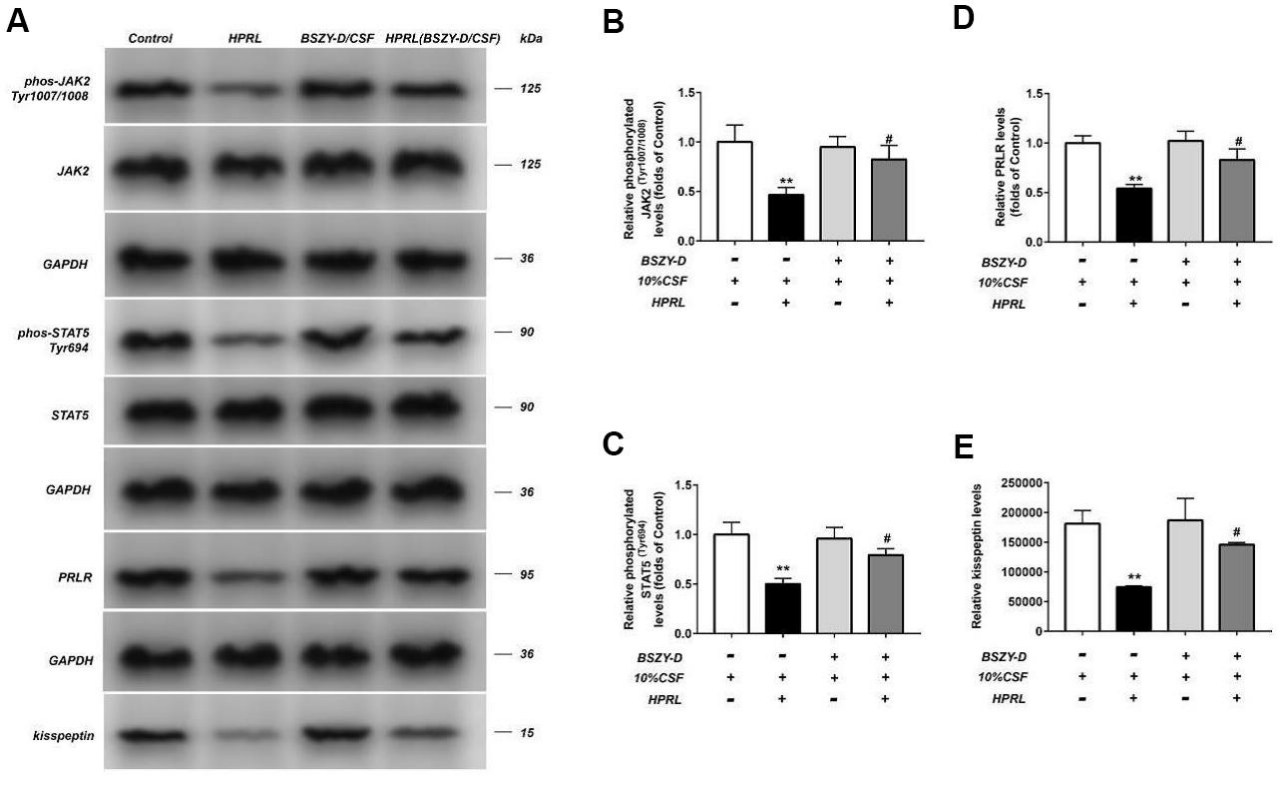
**Effect of CSF on PRLR-JAK-STAT5 pathway in GT1-7.** The phosphorylation levels of JAK, STAT5 and the levels of PRLR and kisspeptin in GT1-7 from different groups were detected by western blot assay, and representative bands were shown in (**A**). The phosphorylation levels of JAK (**B**) and STAT5 (**C**), and the level of PRLR (**D**), and the level of kisspeptin (**E**) were normalized to control. The results were presented as mean ± SD (n = 3).**p < 0.01 vs. Control group, #p < 0.05, ##p < 0.01 vs. HPRL group.

The phosphorylation levels of JAK, STAT5, and the levels of PRLR and kisspeptin in different groups were detected by western blot (Figure 2). Our data indicated that compared with the Control group, the phosphorylation levels of JAK2 and STAT5, and the levels of PRLR and kisspeptin in the HPRL group were decreased significantly (P < 0.05). Compared with the HPRL group, the phosphorylation levels of AK2 and STAT5, and the levels of PRLR and kisspeptin in the HPRL (BSZY-D/CSF) group were significantly elevated (P < 0.05), indicating that BSZY-D reversed the impact of HPRL on the downregulation of the PRLR/JAK/STAT5 pathway.

Similarly, the kisspeptin mRNA expression in the HPRL group was significantly decreased, however, the PRLR mRNA expression was not downregulated significantly; compared with the HPRL group, the kisspeptin mRNA expression in the HPRL (BSZY-D/CSF) group was significantly increased, suggesting that BSZY-D reversed the downregulation trend of kisspeptin through HPRL (Supplementary Figure 4).

### The changes of PRLR expression in GT1-7 cells treated with different reagents

**Figure 3:**
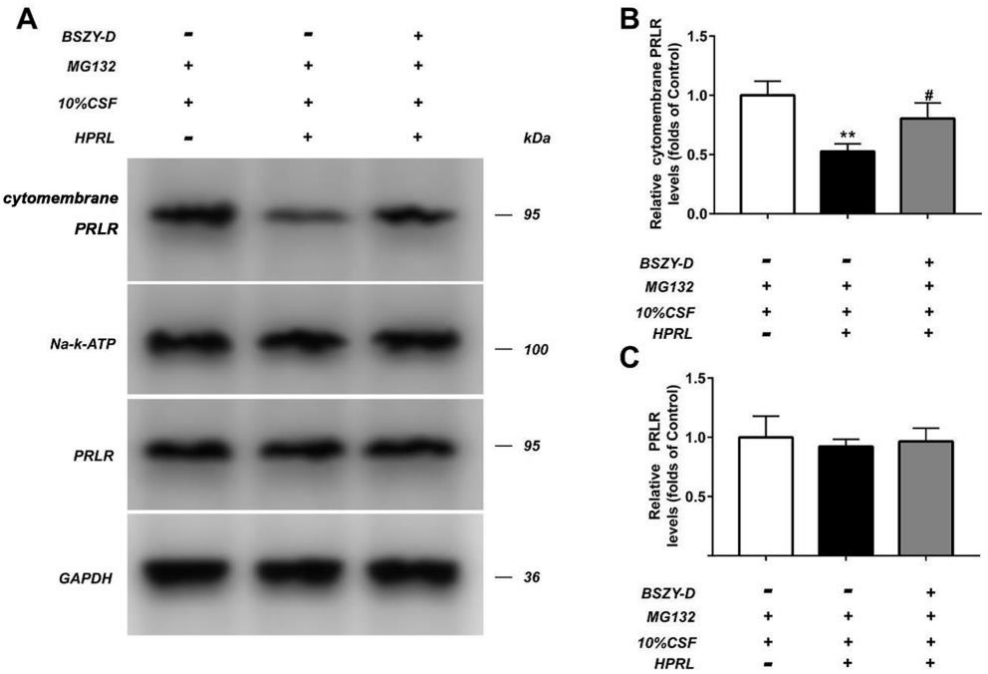
The level of PRLR in GT1-7 from different groups was detected by western blot assay, and representative bands were shown in (**A**). The levels of PRLR (**B**, **C**) were normalized to control. The results were presented as mean ± SD (n = 3). **p < 0.01 vs. Control group, #p < 0.05, ##p < 0.01 vs. HPRL group.

The level of PRLR in different GT1-7 cell groups were detected by western blot assay. As shown in Figure 3, compared with the Control group, the cytomembrane PRLR level in the HPRL group was significantly inhibited, but the total protein level of PRLR remained stable. Compared with the HPRL group, the protein level of PRLR in the HPRL (BSZY-D/CSF) group was significantly upregulated, which suggested that BSZY-D reversed the downregulation of PRLR protein level via HPRL (P < 0.05). Besides, we found that the PRLR ubiquitination level was increased in the HPRL group, however, there was deubiquitination of PRLR in the HPRL (BSZY-D/CSF) group (P < 0.05). It was indicated that HPRL caused the PRLR ubiquitination, but BSZY-D induced the deubiquitination of PRLR (Supplementary Figure 5).

**Figure 4:**
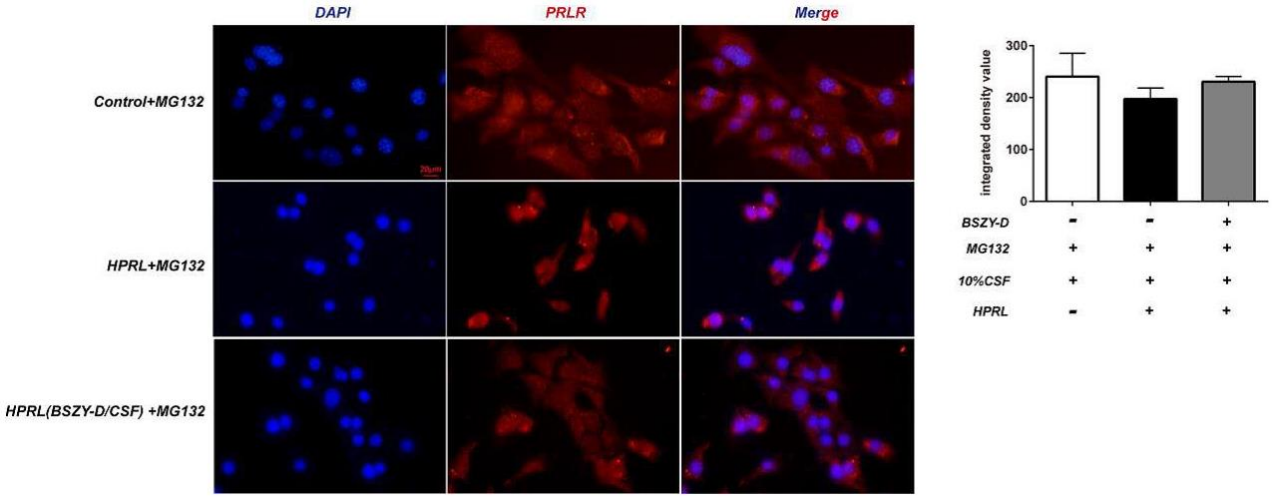
The immunofluorescence staining of PRLR in GT1-7.

Compared with the Control group, the PRLR protein level was downregulated in the HPRL group, but there was no difference in total protein level. Moreover, compared with the HPRL group, the PRLR protein level was augmented in the HPRL (BSZY-D/CSF) group, suggesting that CSF reversed the downregulation of PRLR protein level through HPRL (P < 0.05) (Figure 4).

**Figure 5:**
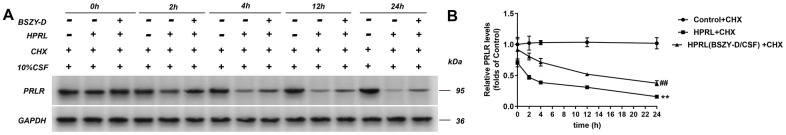
**The level of PRLR in GT1-7 at different times was detected by western blot assay.** The levels of PRLR were normalized to control. (**A**) Representative blot of PRLR level. (**B**) Quantitative relative gray values of PRLR protein level. The results were presented as mean ± SD (n = 3). **p < 0.01 vs. Control group, ##p < 0.01 vs. HPRL group.

The PRLR protein level of GT1-7 cells at different time points were detected using western blot. Compared with the Control + CHX group, the PRLR protein level was decreased significantly in the HPRL group. Compared with the HPRL + CHX group, the PRLR level in the HPRL (BSZY-D/CSF) + CHX group was significantly upregulated (P < 0.05). The results demonstrated that the PRLR degradation would be accelerated under the high PRL condition, and this degradation was inhibited after the addition of BSZY-D (Figure 5).

### The changes of CSN5 expression in GT1-7 cells treated with different reagents

Co-IP was applied to test the expressions of β-Trcp, PRLR and CSN5 (Supplementary Figure 6). Compared with the Control group, the binding of PRLR and β-Trcp was significantly increased in the HPRL group, and the binding of PRLR and CSN5 was decreased; compared with the HPRL group, the binding of PRLR and CSN5 in the HPRL (BSZY-D/CSF) group was increased (P < 0.05). It was suggested that BSZY-D played a role by promoting the binding of PRLR and CSN5.

**Figure 6:**
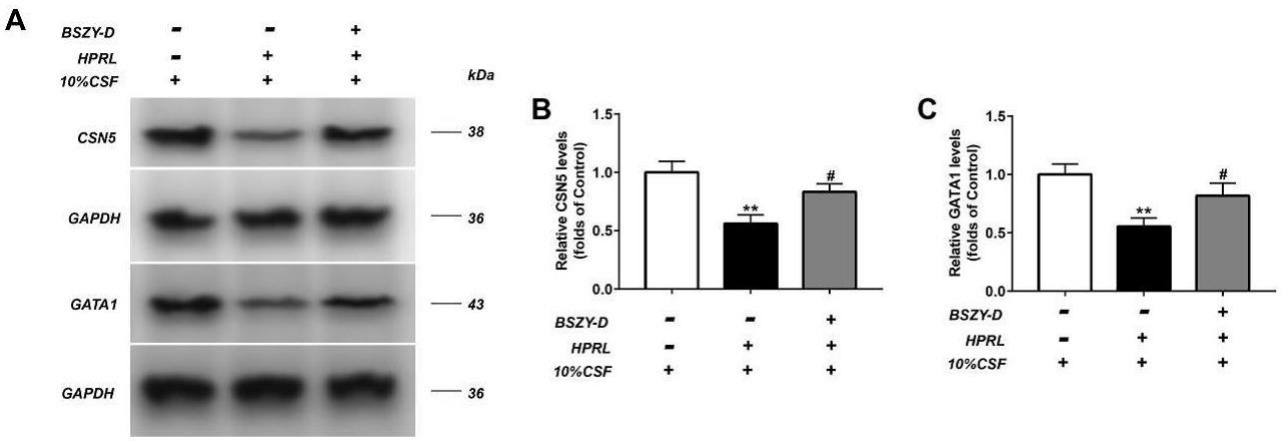
The levels of CSN5 and GATA1 in GT1-7 were detected by western blot assay, and representative bands were shown in (**A**). The levels of CSN5 (**B**) and GATA1 (**C**) were normalized to control. The results were presented as mean ± SD (n = 3).**p < 0.01 vs. Control group, #p < 0.05, ##p < 0.01 vs. HPRL group.

The levels of CSN5 and GATA1 in GT1-7 were detected by western blot (Figure 6). Our results represented that the CSN5 and GATA1 protein levels were significantly decreased in the HPRL group compared with the Control group. Compared with the HPRL group, the levels of CSN5 and GATA1 in the HPRL (BSZY-D/CSF) group were significantly elevated (P<0.05). In line with the results of western blot, the RT-PCR data showed that the CSN5 mRNA expression was significantly suppressed in the HPRL group compared with the Control group, and the GATA1 mRNA expression did not change significantly. Besides, compared with the HPRL group, the CSN5 mRNA expression was significantly increased in the HPRL (BSZY-D/CSF) group (P<0.05) (Supplementary Figure 7). It was indicated that BSZY-D reversed the downregulation effect of HPRL on CSN5.

### Effect of lentivirus plasmids shRNA-CSN5 on PRLR/JAK/STAT5 pathway in GT1-7

**Figure 7:**
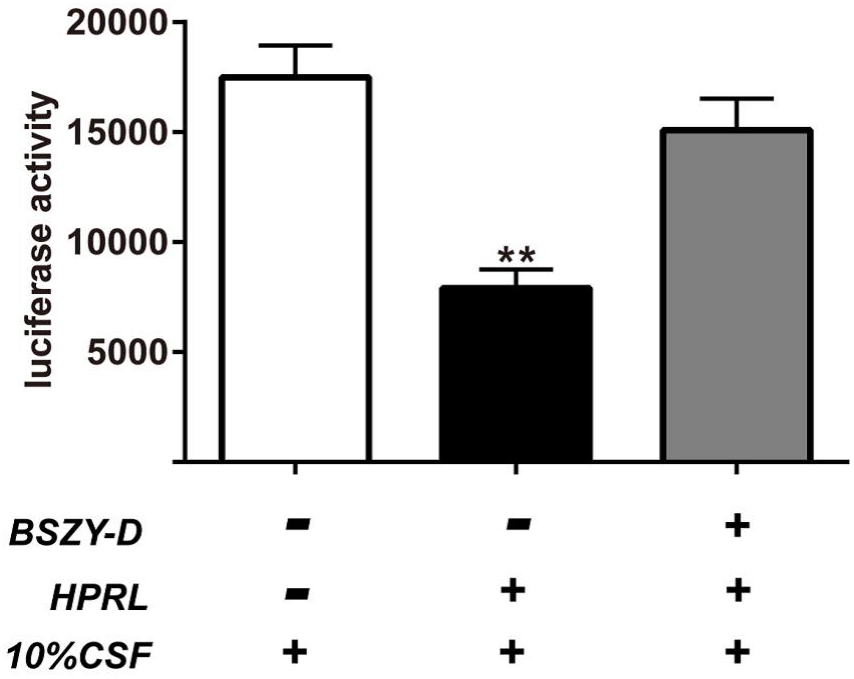
**Luciferase activity measured and normalized according to renilla luciferase activity in GT1-7 cells transiently transfected with the CSN5 luciferase promoter.** The results were presented as mean ± SD(n = 3). **p < 0.01 vs. Control group, ##p < 0.01 vs. HPRL group.

Luciferase activity was detected by measuring the renilla luciferase activity of GT1-7 cells transiently transfected with the CSN5 luciferase promoter (Figure 7). Compared with the Control group, the CSN5 promoter activity in the HPRL group was significantly decreased; in turn, compared with the HPRL group, the CSN5 promoter activity in the HPRL (BSZY-D/CSF) group was significantly upregulated. Correspondingly, the binding of GATA1 and CSN5 promoter in the HPRL group was significantly decreased, but the binding of GATA1 and CSN5 promoter in the HPRL (BSZY-D/CSF) group was significantly elevated (P < 0.05) (Supplementary Figure 8). After the transfection of lentivirus plasmids, the CSN5 protein level was significantly inhibited in the shRNA-CSN5 group, suggesting that the shRNA-CSN5 plasmid reduced the expression of CSN5 in GT1-7 cells (P < 0.05) (Supplementary Figure 9).

**Figure 8:**
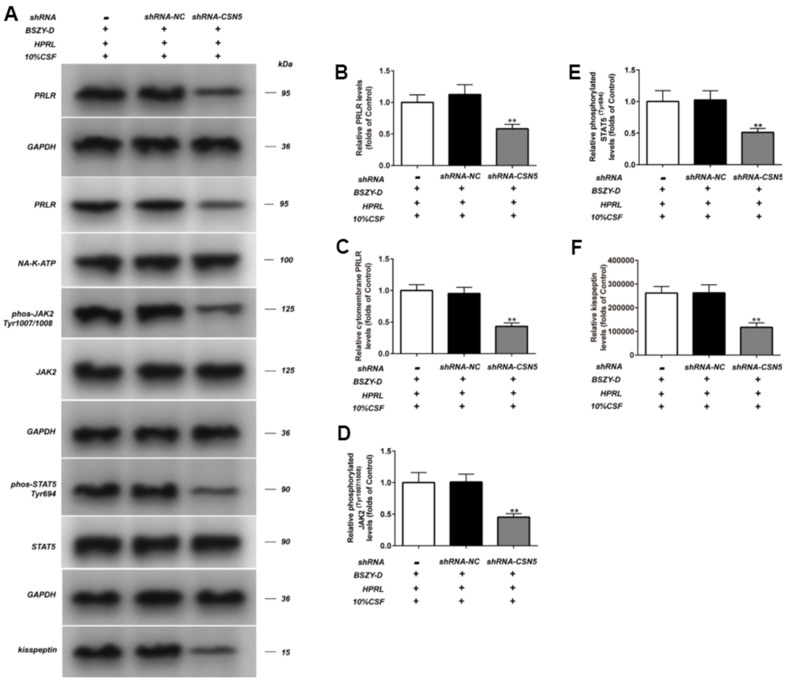
**Effect of shRNA-CSN5 on PRLR-JAK-STAT5 pathway in GT1-7.** The phosphorylation levels of JAK, STAT5 and the levels of PRLR and kisspeptin in GT1-7 from different groups were detected by western blot assay, and representative bands were shown in (**A**). The phosphorylation levels of JAK (**D**) and STAT5 (**E**), and the level of PRLR (**B**, **C**), and the level of kisspeptin (**F**) were normalized to control. The results were presented as mean ± SD (n = 3). *p < 0.05, **p < 0.01 vs. Control group.

After the confirmation of transfection efficiency, the impact of shRNA-CSN5 on PRLR/JAK/STAT5 signaling pathway in GT1-7 cells of different treatment groups was detected by western blot. As shown in Figure 8, compared with the Control group, the phosphorylation levels of JAK2 and STAT5, and the protein levels PRLR and kisspeptin in the HPRL (BSZY-D/CSF) + shRNA-NC group were significantly decreased (P < 0.05). After knocking down CSN5 gene, the above-mentioned protein levels could be reversed. This result indicated that BSZY-D enhanced the PRLR/JAK/STAT5 signaling pathway through CSN5.

Besides, there existed an increasing ubiquitination level of PRLR in the HPRL group, which could be reversed by knocking down shRNA-SCN5. It was suggested that BSZY-D played a role through targeting CSN5 (Supplementary Figure 10).

## DISCUSSION

**Figure 9:**
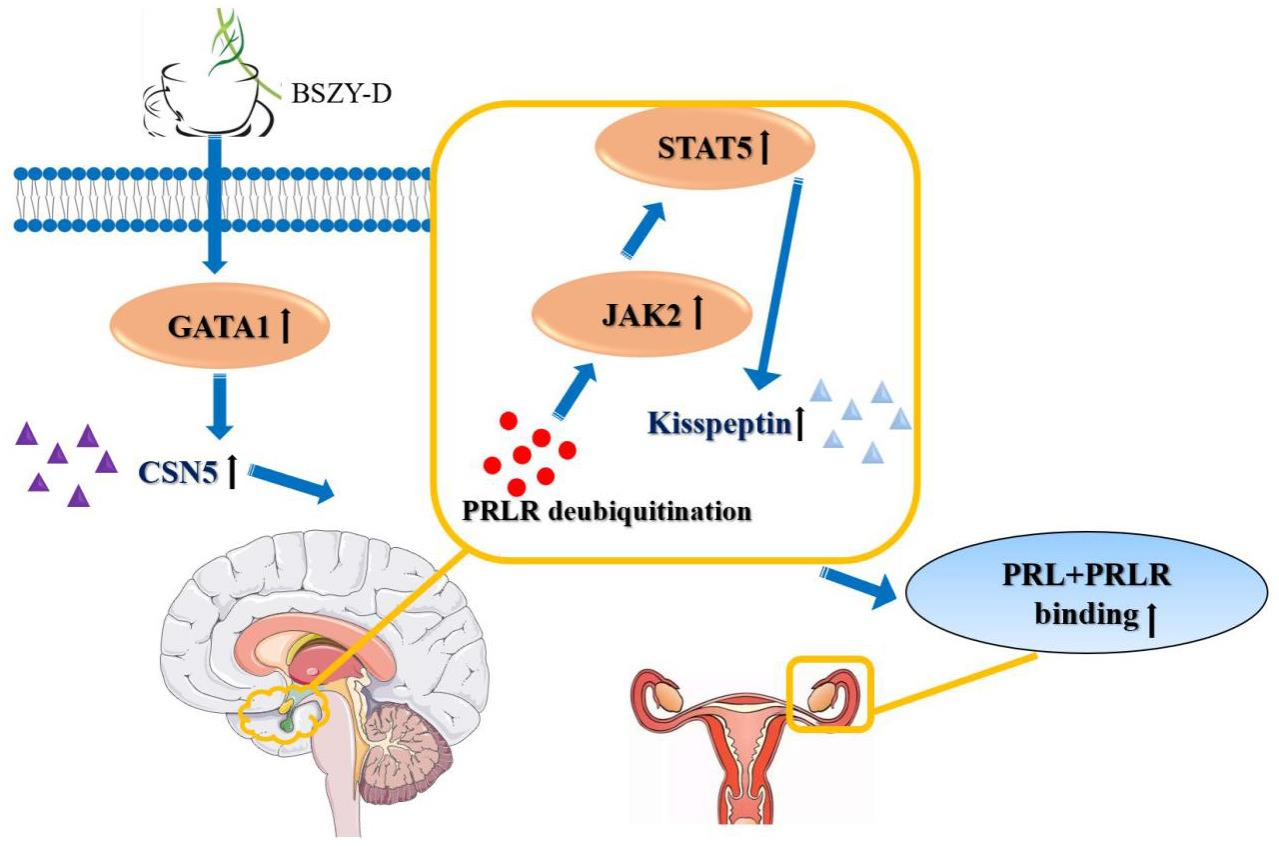
**The effects of BSZY-D on activating GATA 1 and CSN5, inducing PRLR deubiquitination and increasing the expressions of JAK2/STAT5 signaling pathway and kisspeptin.** Hence, BSZY-D had an efficient role in HPRL infertility improvement.

Previous researches of traditional Chinese medicine Bu-Shen-Zhu-Yun Decoction (BSZY-D) mainly focus on infertility caused by luteal phase defect. Recently, it is reported that BSZY-D can reduce the prolactin (PRL) level in patients with luteal phase defect and hyperprolactinemia (HPRL) in clinic practice. Hence, our purpose in this study is to investigate the underlying mechanism of BSZY-D in decreasing PRL. In this study, we identified that HPRL could downregulate the levels of PRLR and kisspeptin, and phosphorylation levels of JAK2 and STAT5. BSZY-D reversed the effect of HPRL on the downregulation of the PRLR/JAK2/STAT5 pathway. The potential mechanism was that excessive PRL cannot bind to its receptor under high PRL microenvironment, thus the expressions of JAK2/STAT5 pathway and kisspeptin were suppressed. Besides, CSN5 inhibited the degradation of PRLR through deubiquitination, and targeted its transcription factors GATA 1. These changes could be reversed after BSZY-D treatment. We proved that BSZY-D increased the binding level of CSN5 and GATA 1, and induced the deubiquitination of PRLR, thus increasing the expressions of JAK2/STAT5 pathway and its downstream signaling kisspeptin. Based on our study, BSZY-D can be regarded as an effective Chinese medicine to treat HPRL infertility in clinic (Figure 9).

The theory of Chinese medicine believes that the kidney affects reproduction, which is the innate foundation and the root of vitality. Ovulation relies on several factors, such as kidney, liver, spleen, qi, blood, yin and yang [7]. A previous study of meta-analysis has represented that BSZY-D can significantly elevate the pregnancy rate to 50%, comparing to the pregnancy rate of 30% using vitro fertilization [11]. Several ingredients of BSZY-D including dioscoreae rhizome, cervi cornu, bupleuri radix and paeoniae radix alba, etc. have been proved to improve the uterine receptivity and pregnancy rate in mice [12, 13]. Xiaofei Jiang et al. have demonstrated that BSZY-D can upregulate the expressions of integrin α5 and β3 by inhibiting the estrogen and progesterone receptor, meanwhile, improve the endometrial receptivity during embryo implantation and consequently enhance the pregnancy rates [14]. In the current study, we found that BSZY-D reserved the molecular and pathway changes caused by HPRL. The elevation of PRLR, kisspeptin, phosphorylated JAK2, and STAT5 levels were significantly reserved by BSZY-D treatment, as well as the decline of the PRLR ubiquitination.

Prolactin (PRL) is a type of polypeptide hormone secreted by the anterior pituitary gland. PRL is balanced by PRL releasing factor (PRF) and PRL inhibiting hormone (PIH) [15, 16]. In the normal physiological condition, the increased transcription activity of GATA1 induces the transcription of its downstream CSN5 gene, leading to the upregulation of CSN5. CSN5 plays a role in mediating the deubiquitination of PRLR, blocking its endocytosis and degradation, and maintaining the normal function of PRLR. JAK2/STAT5 is the downstream signaling pathway of PRLR, and the activation of JAK2/STAT5 can elevate the expression of kisspeptin. In the presence of pathological HPRL condition, HPRL can reduce the receptivity of the endometrium, and suppress the ovarian function, consequently causing ovulation disorders [17]. Recently, it is reported that the proper concentration of PRL can promote the growth of endometrial cells and blastocyst adhesion. However, the deregulated PRL concentration has an inhibitory effect, which causes follicle maturation disorder, ovulation disorder and decreased endometrial receptivity, thus eventually leading to infertility [18]. The underlying mechanism can be explained by an abnormal level of PRLR ubiquitination. The overexpressing PRL augments the activity of CSN5, a type of deubiquitinating enzyme, which induces the deubiquitination of PRLR. Hence, the downstream JAK2/STAT5 signaling pathway is inhibited, as well as kisspeptin. In the current study, we identified that BSZY-D could reversely elevate the transcription activity of GATA1, increase the binding of GATA1 and CSN5, and then reserve the downstream expressions at a certain level.

Kisspeptin is a type of polypeptide encoded by the KISS-l gene. It is mainly distributed in the hypothalamus, pituitary, ovary, placenta, etc. Recent studies have represented that kisspeptin plays an important role in the initiation of puberty, menstrual cycle regulation, ovulation induction, and reproductive cycle regulation [19]. Some scholars have indicated that kisspeptin may have a certain correlation with pregnancy implantation, and further hypothesized it is also related to abortion [20]. In the current study, our results showed that HPRL could downregulate the expression of kisspeptin, which might impair the physiological role of kisspeptin in maintaining the normal productive function. Interestingly, kisspeptin expression could be reversed after BSZY-D treatment. Besides, in line with the changes of kisspeptin, the expression of PRLR/JAK/STAT5 signaling pathway showed the similar trend.

In this study, we proved that BSZY-D partly recovered the expressions of JAK/STAT5 and kisspeptin via inducing the deubiquitination of PRLR and targeting CSN5. The limitation of this study is that we did not clarify the possible molecular mechanism between CSN5 and PRLR deubiquitination, which will be investigated as a key point in our further study.

## CONCLUSIONS

Altogether, we explored the underlying mechanism of BSZY-D in HPRL infertility. We demonstrated that BSZY-D could improve the transcription activity of GATA1 and increase the binding of GATA1 and CSN5. Also, the activated deubiquitinating enzyme CSN5 could increase the expressions of JAK/STAT5 and kisspeptin via inducing the deubiquitination of PRLR, thereby alleviating the clinic manifestations of HPRL infertility. We hope our novel work could establish a theoretical basis on the molecular mechanism of BSZY-D in HPRL infertility, meriting further investigation of potential treatment application.

## MATERIALS AND METHODS

### Cell culture and treatment

GT1-7 cell line was purchased from Millipore (Schwalbach, Germany). GT1-7 cells were cultured and maintained in DMEM medium (GIBCO, Grand Island, USA) containing penicillin (final concentration 100U/mL) (Sigma-Aldrich, St. Louis, MO, USA), streptomycin (final concentration 100μg/mL) (Sigma-Aldrich, St. Louis, MO, USA), 2mM L-Glutamine (Millipore, Schwalbach, Germany) and 10% fetal bovine serum (FBS) (Hyclone, Logan, UT, USA). The cells were digested when reaching 90% confluence.

### Medicine and extraction preparation

BSZY-D was concentrated to 2g/mL and stored at 4° C. In brief, the medicinal materials had been immersed in water for 30 min. Firstly, the deer horn slices were decocted and boiled for 20 min, and then added with the remaining medicinal materials. The decoction at a concentration of 2 g/mL was collected and stored at 4° C.

A total of 75 female SD rats of specific pathogen-free (SPF) grade (aged 8 weeks and weighing 220 ± 20 g) were purchased from Animal Model Center of Nanjing University [license No: SCXK (Su) 2018-0008], and raised in the SPF environment in the Experimental Animal Center of Nanjing Chinese Medicine University. The rats were reared at 40%-70% humidity and 21-25° C, maintained in a 12 h light/dark cycle. The experimental operations were approved by the Animal Ethics Committee of Nanjing Chinese Medicine University [approval No: SYXK (Su) 2014-0001].

Cerebrospinal fluid was prepared as follows: BSZY-D of 2 g/mL was given to normal female SD rats 1 mL/kg twice a day. After anesthesia, the neck skin of each rat was disinfected with 75% ethanol, and the foramen magnum was exposed. The micro-injector was pierced into the cistern magna and 100 uL cerebrospinal fluid (CSF) was slowly extracted. After centrifugation at 2000 r/min for 15 min, the supernatant was collected and stored at -20° C. The time point of CSF collection was after the estrus.

### Experimental groups

Protocols in different experimental groups were shown as follows:

1) Experiment 1: 0% CSF (medicated cerebrospinal fluid), 5% CSF (medicated cerebrospinal fluid), 10% CSF (medicated cerebrospinal fluid) and 20% CSF (medicated cerebrospinal fluid).

2) Experiment 2: 0 ng/mL, 12.5 ng/mL, 25 ng/mL, 50 ng/mL, 100 ng /mL, 200 ng/mL and 400 ng/mL PRL.

3) Experiment 3: 0 ng/mL, 12.5 ng/mL, 25 ng/mL, 50 ng/mL, 100 ng /mL and 200 ng/mL PRL.

4) Experiment 4:

Normal group (Control group): 10% blank cerebrospinal fluid, cultured for 72 h.

PRL intervention group (HPRL group): 10% blank cerebrospinal fluid + 200 ng/mL PRL, cultured for 72 h.

Cerebrospinal fluid containing BSZY-D intervention group (BSZY-D/CSF group): 10% BSZY-D containing medicated cerebrospinal fluid, cultured for 72 h.

Cerebrospinal fluid containing traditional Chinese medicine at a concentration of 10% [HPRL (BSZY-D/CSF) group]: 10% BSZY-D containing cerebrospinal fluid culture + 10% blank cerebrospinal fluid + 200 ng/mL PRL, cultured for 72h.

5) Experiment 5:

Normal group + MG132 (Selleck Chemicals, Houston, Texas, USA) (Control + MG132 group): 10% blank cerebrospinal fluid + 10 uM MG132 inhibitor, cultured for 72h.

PRL intervention group + MG132 (HPRL + MG132 group): 10% blank cerebrospinal fluid + 200 ng/mL PRL + 10 uM MG132 inhibitor, cultured for 72h.

Cerebrospinal fluid containing traditional Chinese medicine a concentration of 10% + MG132 [HPRL (BSZY-D/CSF) group]: 10% BSZY-D containing cerebrospinal fluid culture + 200ng/mL PRL + 10uM MG132 inhibitor, cultured for 72h.

6) Experiment 6:

Normal group + cycloheximide (CHX) (Seleck Chem, Shanghai, China) (Control + CHX group): 10% blank cerebrospinal fluid + 10uM CHX inhibitor.

PRL intervention group + cycloheximide CHX (HPRL + CHX group): 10% blank cerebrospinal fluid + 200 ng/mL PRL + 10uM CHX inhibitor.

Cerebrospinal fluid containing traditional Chinese medicine at a concentration of 10% + cycloheximide CHX [HPRL (BSZY-D/CSF) + CHX group]: 10% BSZY-D containing medicated cerebrospinal fluid culture + 200 ng/mL PRL + 10 uM CHX inhibitor.

The time points for detection were 0h, 2h, 4h, 12h, 24h, respectively.

7) Experiment 7:

Normal group + MG132 (Control + MG132 group): 10% blank cerebrospinal fluid + 10 uM MG132 inhibitor, cultured for 72h.

PRL intervention group + MG132 (HPRL + MG132 group): 10% blank cerebrospinal fluid + 200 ng/mL PRL + 10 uM MG132 inhibitor, cultured for 72h.

Cerebrospinal fluid containing traditional Chinese medicine at a concentration of 10% + MG132 [HPRL (BSZY-D/CSF) +MG132 group]: 10% BSZY-D containing medicated cerebrospinal fluid culture + 200 ng/mL PRL + 10 uM MG132 inhibitor, cultured for 72h.

8) Experiment 8:

Normal group (Control group): 10% blank cerebrospinal fluid, cultured for 72 hours.

Prolactin intervention group (HPRL group): 10% blank cerebrospinal fluid + 200 ng/mL PRL, cultured for 72 h.

Cerebrospinal fluid containing traditional Chinese medicine a concentration of 10% intervention [HPRL (BSZY-D/CSF) group]: 10% BSZY-D containing medicated cerebrospinal fluid culture + 200 ng/mL PRL, cultured for 72h.

9) Experiment 9:

Cerebrospinal fluid containing traditional Chinese medicine at a concentration of 10% intervention [HPRL (BSZY-D/CSF) group]: 10% BSZY-D containing medicated cerebrospinal fluid culture + 200 ng/mL PRL, cultured for 72h.

Cerebrospinal fluid containing traditional Chinese medicine a concentration of 10% + ShRNA-CSN5 [HPRL (BSZY-D/CSF) + ShRNA-CSN5 group]: after the cells were stably transfected with CSN5-knockdown lentivirus, added 10% BSZY-D containing medicated cerebrospinal fluid culture + 200 ng/mL PRL, cultured for 72h.

Cerebrospinal fluid containing traditional Chinese medicine a concentration of 10% + ShRNA- NC [HPRL (BSZY-D/CSF) + ShRNA-NC group]: after the cells were stably transfected with NC lentivirus, added 10% BSZY-D containing medicated cerebrospinal fluid culture + 200 ng/mL PRL, cultured for 72h.

### CCK8 assay

The GT1-7 cell density was adjusted to 5000 cells/well, seeded into a 96-well plate, and cultured in the incubator at 37° C with 5% CO_2_ for 24 h. Afterwards, the original culture solution was discarded and the cells were washed twice with phosphate-buffered saline (PBS). According to Experiment 1 or 2, GT1-7 cells were treated differently. Each experiment group had 3 parallel wells. After 72h of incubation, each well was added with 10 μL CCK-8 (Beyotime Biotechnology, Shanghai, China) solution and incubated for 2 h in an incubator. The optical density (OD) value of the liquid in each well was measured by a microreader (Berthold LB941) at the wavelength of 450 nm.

### Western blot

Total protein was extracted from GT1-7 cells using RIPA lysis buffer (Beyotime Biotechnology, Shanghai, China). The tissue lysates were centrifuged at 12,000 rpm for 5 min at 4° C, and the supernatants were collected for further analysis. The bicinchoninic acid (BCA) assay was applied to detect the concentration of protein samples. Then, 60 μg protein samples per lane were separated using 8% SDS-PAGE and then transferred onto PVDF membranes (0.45 μm, Millipore, Schwalbach, Germany). The membranes were blocked in Tris-buffered saline Tween containing 5% skim milk for 1h at room temperature and then incubated overnight at 4° C with primary antibodies: Anti-phos-JAK2 (Tyr1007/1008) antibody (3771), Anti-JAK2 antibody (3230), Anti-phos-STAT5 (Tyr694) antibody (9314), Anti-STAT5 antibody (94205), Anti-NA/K ATP antibody (3010) (CST, Boston, USA); Anti-PRLR antibody (ab170935), Anti-kisspeptin antibody (ab19028) (Abcam, Cambridge, UK); Anti-PRLR antibody (ABIN152720) (IP), Anti-GATA1 antibody (ABIN2704828) (antibodies-online, Beijing, China). Next day, these membranes were washed with PBS and incubated with secondary antibodies for 1h at room temperature. After rinsing, the proteins were detected by enhanced chemiluminescence. The protein levels were quantified by densitometry using Image-Pro Plus 6.0 and normalized to the corresponding GAPDH level.

### Real-time PCR

The total RNA was extracted from GT1-7 cells using TRIzol reagent (Invitrogen, Carlsbad, CA, USA). RNA then underwent reverse transcription using the iScript cDNA kit (Bio-Rad, Hercules, CA, USA), followed by analysis using real-time PCR (RT-PCR) with the SsoFast Eva Green Super Mix (Bio-Rad, Hercules, CA, USA) on Roche Light Cycler 480 system.

Relative mRNA expressions of kisspeptin, PRLR, CSN5, GATA1, GAPDH were calculated by the comparative cycle threshold (CT) method, with expression of GAPDH as the internal reference. The 2^−∆∆Ct^ method was used for analysis. The specific primers applied for RT-PCR reaction are shown in Table 1.

**Table 1:** Primers used in quantitative real-time PCR and shRNA-CNS5 sequence.

**Primers**	**Sequences (5’→3’)**
**kisspeptin**	F- CTCTGTGTCGCCACCTATGG R- AGGCTTGCTCTCTGCATACC
**PRLR**	F- GACTCAAGGGGGCAAAGTCA R- CACCTCCACAGAGAAGCGTT
**CSN5**	F- GCCTTGAGAGTCTATCACCACT R- TGATGATCATGGTCTCGCCG
**GATA1**	F- TTGGGATCACCCTGAACTCG R- GGTTGAACCTGGGCTTGTTG
**GAPDH**	F- CATCACTGCCACCCAGAAGACTG R- ATGCCAGTGAGCTTCCCGTTCAG
**ShRNA-CSN5**	CCGGAGCGCAGAGTATCGATGAAATCTCG AGATTTCATCGATACTCTGCGCTTTTTTG

### Co-immunoprecipitation (Co-IP)

The GT1-7 cells were treated as described in Experiment 5, 7 and 9. Then the cell precipitation was added with RIPA lysis buffer. Protein A/G-agarose microspheres were washed twice with PBS, and prepared into 50% protein A/G-agarose working solution with PBS. Next, 50% protein A/G agarose working solution was added into the samples at a ratio of 100 μL per 1 mL. The protein A/G-agarose microspheres were discarded by centrifugation. BCA assay was applied to test the protein concentration. Afterwards, 5 μL antibody (PRLR150) (IgG antibody as the control group) was added for incubation at 4° C overnight and the final volume was 500 μL. Next day, 100 μL protein A/G-agarose microspheres was added to capture the antigen-antibody complex at room temperature for 1h. The protein A/G-agarose microsphere antigen-antibody complex was collected after centrifugation and washed with PBS. The supernatant was collected, and the loaded samples were boiled for 5 min to precipitate. The obtained samples were detected by western blot using antibodies (β-Trcp, 4394, Abcam, Cambridge, UK), as described in 3.4.

### Immunofluorescence

The cells were fixed with 10% paraformaldehyde for 20 min at room temperature. The fixed cells were blocked with 1% bovine serum albumin (BSA) for 30 min and incubated with the corresponding primary antibody (PRLR 1:20) at 4° C overnight, followed by washing with PBST for 10 min. Then the secondary antibody was added for 2h-incubation at room temperature, followed by the 4’, 6-diamidino-2-phenylindole (DAPI) staining for 15 min, and observation under a laser confocal microscope (400 ×).

### Construction of luciferase reporter gene plasmid

The full length of CSN5 (NM001277101) promoter was detected, combining with the target gene prediction software (Jaspar), to find the interaction site of CSN5 promoter and acting site of GATA1. Primer 5 was applied to design primers, making PCR product containing CSN5 promoter and GATA1 predicted binding site 1 (GGATGT).

According to the protocol of Experiment 8, each well of 96-well plate was added with 100 μL reporter gene cell lysate, followed by centrifugation at 15000rpm for 5 min to collect the supernatant. The sample was detected by a microplate reader with an interval of 2 s and a measurement time of 10 s. Then 100 μL luciferase detection reagent was added into 100 μL sample for RLU measurement.

### Chromatin immunoprecipitation assay (ChIP)

The cells were treated with 1% paraformaldehyde and incubated at 37° C for 10min. The cells were resuspended in SDS lysis buffer and broken by ultrasonic, and the supernatant was collected. CHIP diluent was added with protease inhibitor, and this solution was used to dilute the cell supernatant. Then 200 μL cell supernatant was added into 1800 μL ChIP diluent, making the final volume as 2 mL. The 10 μL cell supernatant dilution was regard as input control. Protein A Agarose/salmon sperm DNA was added to the supernatant, followed by antibody (GATA1) incubation overnight. Then 200μL eluent was added to the protein A Agarose/antibody complex, and the supernatant was collected. Next, 20 μL 5MNaCl was added to each 500 μL eluent and subjected to water bath at 65° C for 4h, followed by 10 μL 0.5M EDTA, 20 μL 1M Tris-HC1 and 2 μL proteinase K at a 45° C-water bath for 1h. After filtered by DNA clean-up Column, the fluid was collected, and the qPCR was performed as described as 3.6.

### Construction of lentivirus

Based on the construction of vector and identification of Colony PCR (Supplementary Figure 1), the bacterial solution with correct sequencing was seeded into 10 mL LB liquid medium containing Amp antibiotic and incubated, and then the plasmid was extracted. The GT1-7 cells were seeded into a 24-well plate and prepared for transfection. Then 0.8 μg plasmid was added to 50 μL Opti-MEM medium (GIBCO, Grand Island, USA) or 2.0 μL lipofectamine 2000 (Thermo Fisher Scientific, NY, USA) at room temperature for 5min, respectively. The two diluents were mixed for 5 min, and then 100 μL mixture was added into each well of the cell plate for 12 h-incubation at 37° C. After 72 h of transfection, the infective efficiency could be determined.

### Statistical analysis

All data were expressed as means ± SEM. Assessment of statistical significance was done using statistical analysis software SPSS (IBM SPSS Statistics 21; SPSS Inc., Chicago, IL, USA). For comparisons between experimental groups, one-way ANOVA and Tukey’s test were applied. All the P values were two-sided and the differences were considered statistically significant at P < 0.05.

### Ethical statement

The experimental operations were approved by the Animal Ethics Committee of Nanjing Chinese Medicine University (approval No: SYXK (Su) 2014-0001).

## Supplementary Material

Supplementary Figures
