# Supplementary material for: Bu-Shen-Zhu-Yun decoction induces PRLR deubiquitination and JAK2/STAT5 activation via CSN5 in vitro
Source: Aging (Albany NY). 2021 Aug 23;13(16):20418–37. doi: 10.18632/aging.203426 (PMC8436908; doi:10.18632/aging.203426)
Supplement: Supplementary Figures [file aging-13-203426-s001.pdf]

SUPPLEMENTARY FIGURES

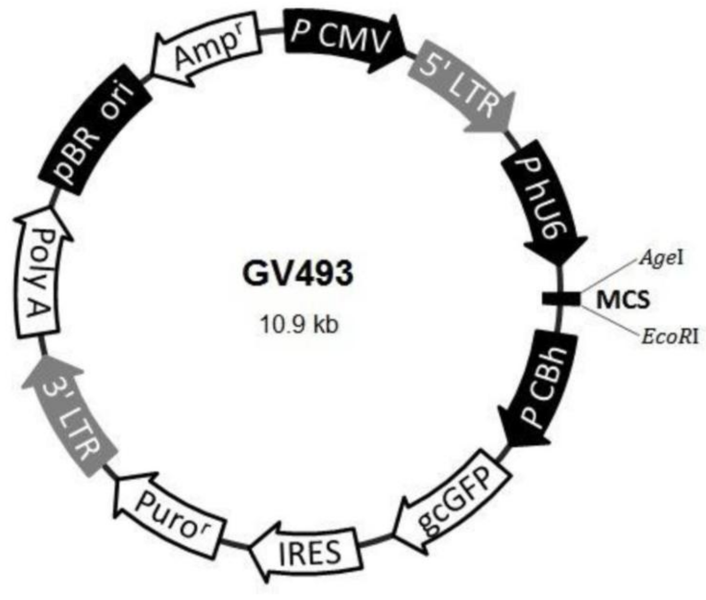

Supplementary Figure 1. The schematic diagram of lentiviral vector.

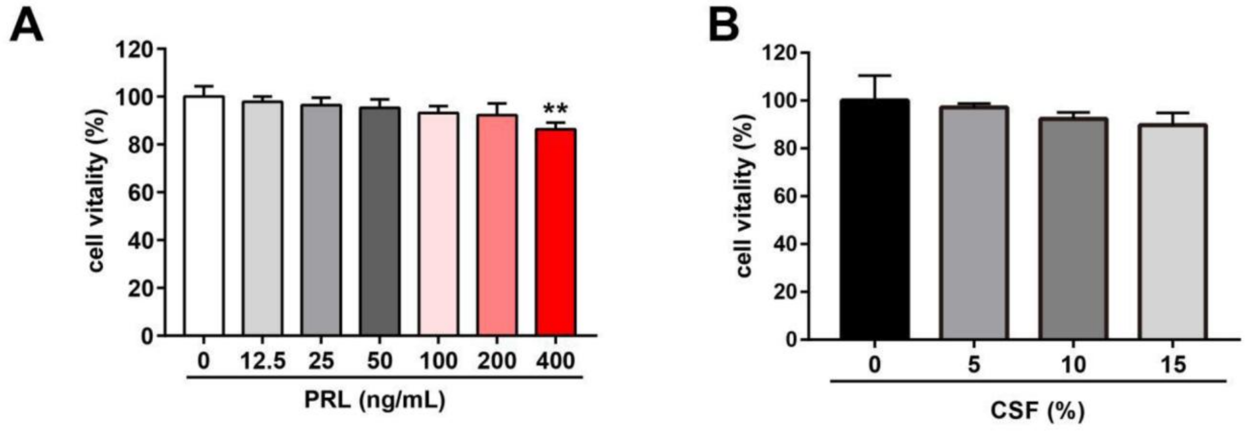

Supplementary Figure 2. Inhibitory effect of PRL or CSF in GT1-7 cells. (**A**) The change of cell vitality effected by different PRL concentration levels. (**B**) The change of cell vitality effected by different CSF concentration levels. Results were expressed as mean ± SD (n=3). **p<0.01 vs Control group.

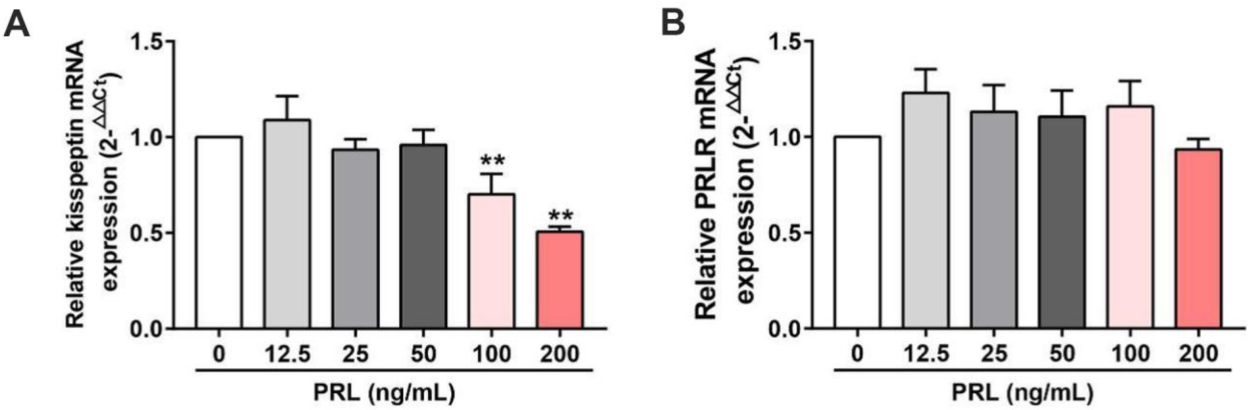

Supplementary Figure 3. Effect of the mRNA expressions of PRLR and kisspeptin in GT1-7. The mRNA expressions of PRLR and kisspeptin were detected by RT-PCR. The mRNA expressions of PRLR (**A**) and kisspeptin (**B**) were normalized to control. The results were presented as mean ± SD (n = 3). **p < 0.01, vs. Control group.

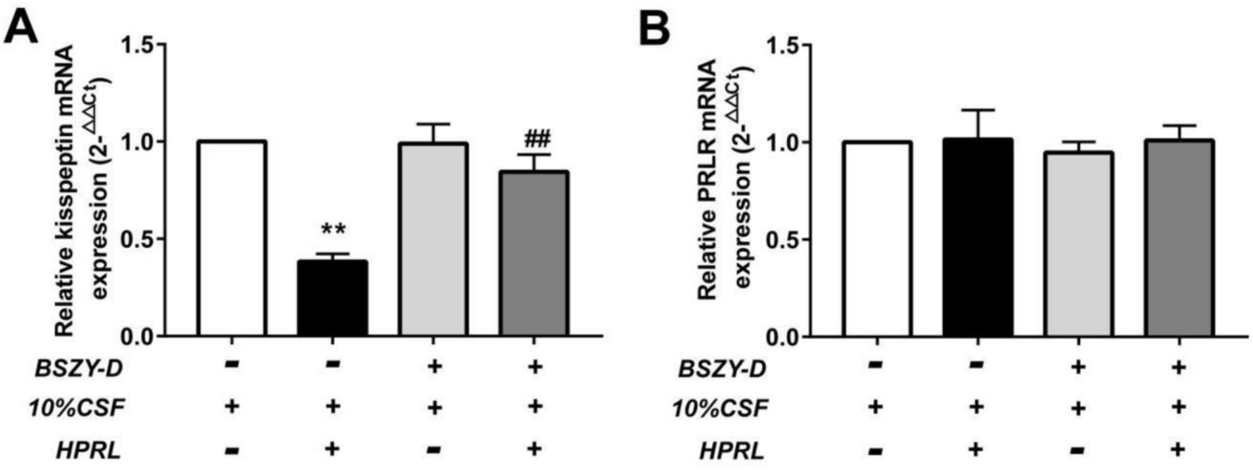

Supplementary Figure 4. Effect of the mRNA expressions of PRLR and kisspeptin in GT1-7. The mRNA expressions of PRLR and kisspeptin were detected by RT-PCR. The mRNA expressions of PRLR (**A**) and kisspeptin (**B**) were normalized to control. The results were presented as mean ± SD(n = 3). **p < 0.01 vs. Control group, ##p < 0.01 vs. HPRL group.

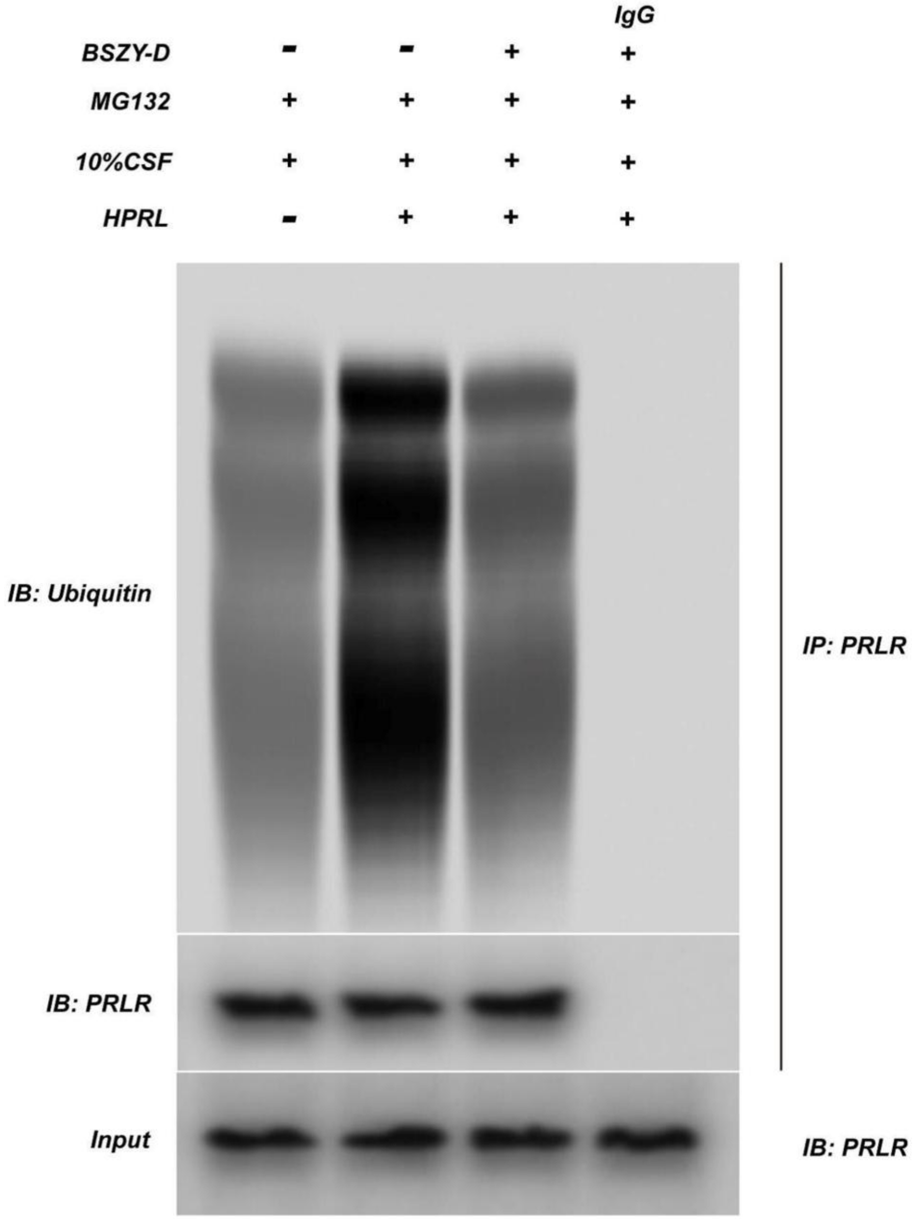

Supplementary Figure 5. The ubiquitin level of PRLR was measured with Co-IP. (n = 3).

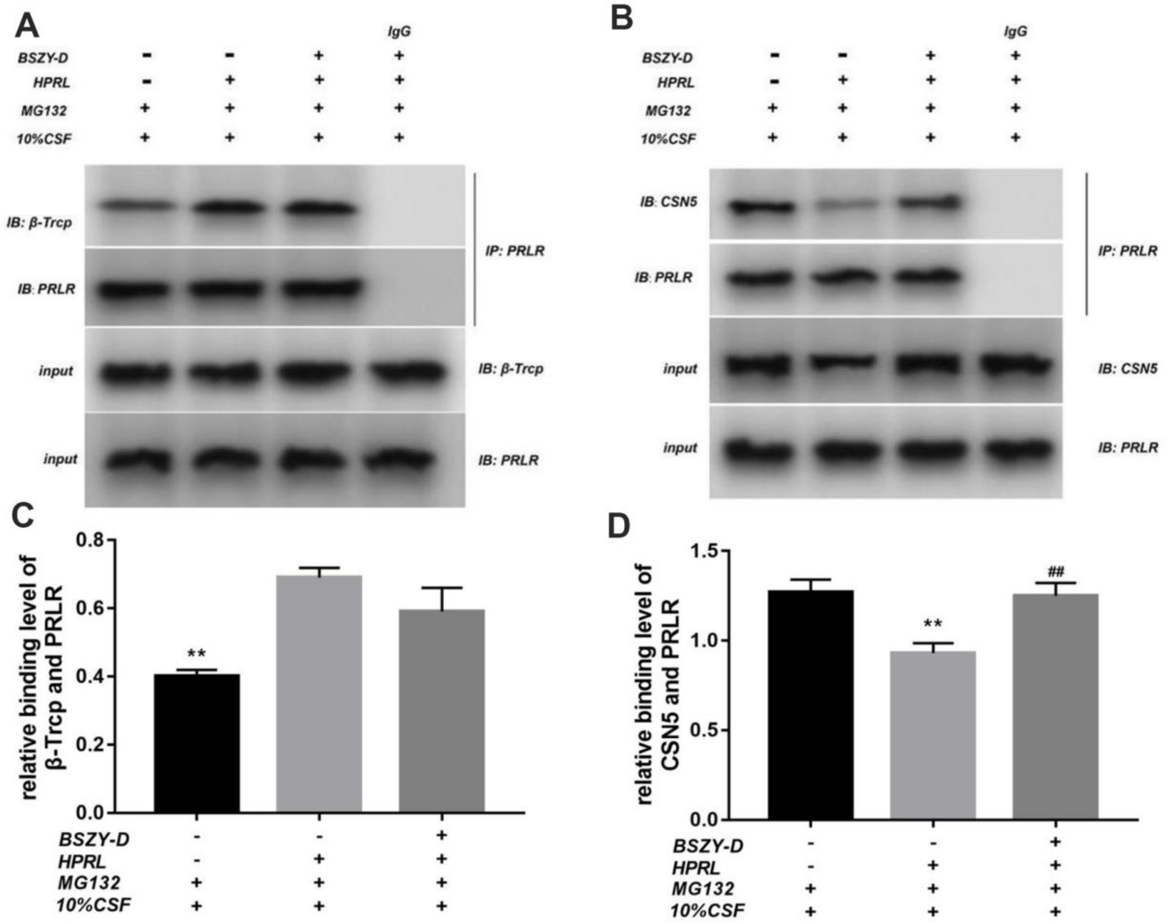

Supplementary Figure 6. The level of β-Trcp, PRLR, and CSN5 were measured with Co-IP (n = 3). (**A**) Representative blots of Co-IP between PRLR andβ-Trcp. (**B**) Representative blots of Co-IP between PRLR and CSN5. (**C**) Quantitative relative binding level of RLR andβ-Trcp. (**D**) Quantitative relative binding level of RLR and CSN5.

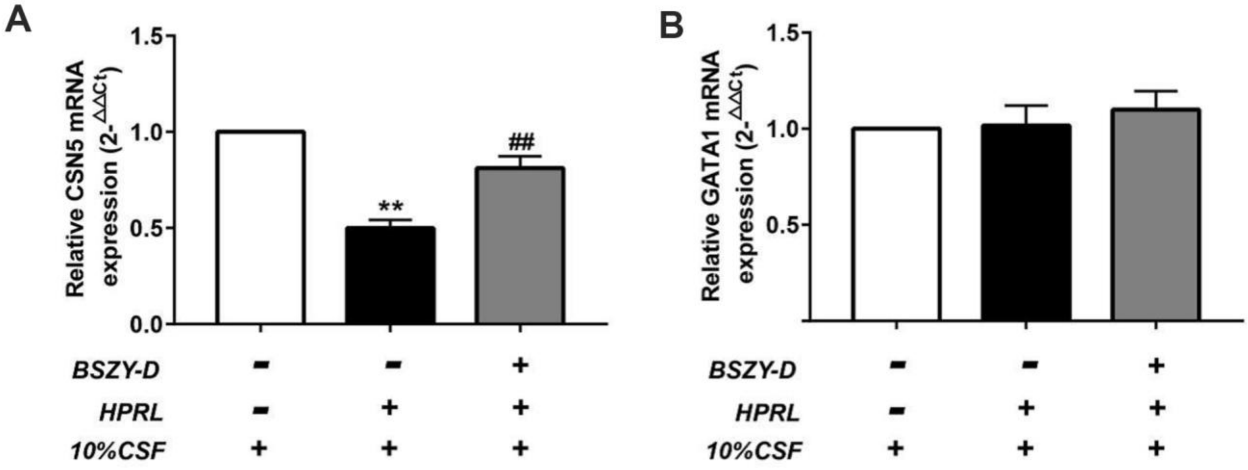

Supplementary Figure 7. Effect of the mRNA expressions of CSN5and GATA1 in GT1-7. The mRNA expressions of CSN5 and GATA1 were detected by RT-PCR. The mRNA expressions of CSN5 (**A**) and GATA1 (**B**) were normalized to control. The results were presented as mean ± SD (n = 3). **p < 0.01 vs. Control group, ##p < 0.01 vs. HPRL group.

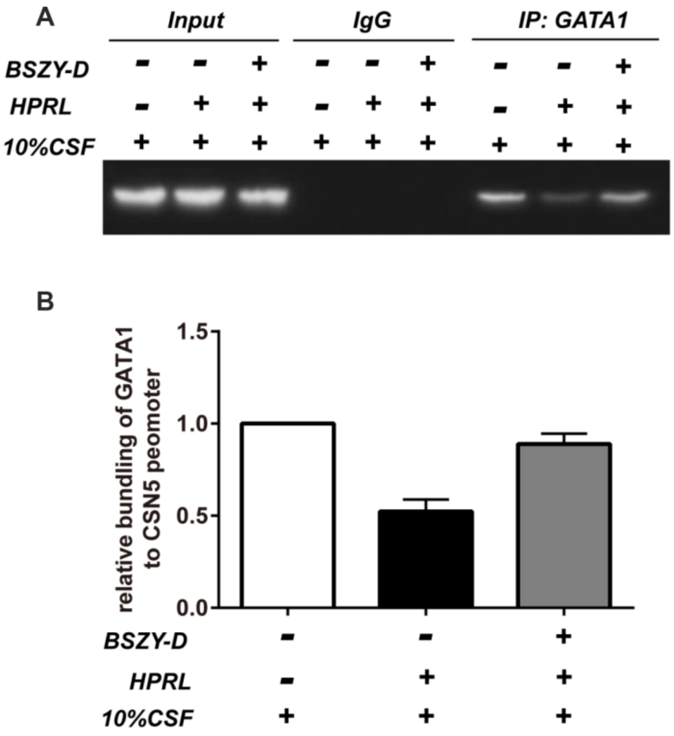

Supplementary Figure 8. Chromatin immunoprecipitation (ChIP) assay on the promoter of GATA1. (**A**) Representative blot of ChIP assay on GATA1. (**B**) Quantitative relative binding of GATA1 to CSN5 promoter. The results were presented as mean ± SD (n = 3). **p < 0.01 vs. Control group, ##p < 0.01 vs. HPRL group.

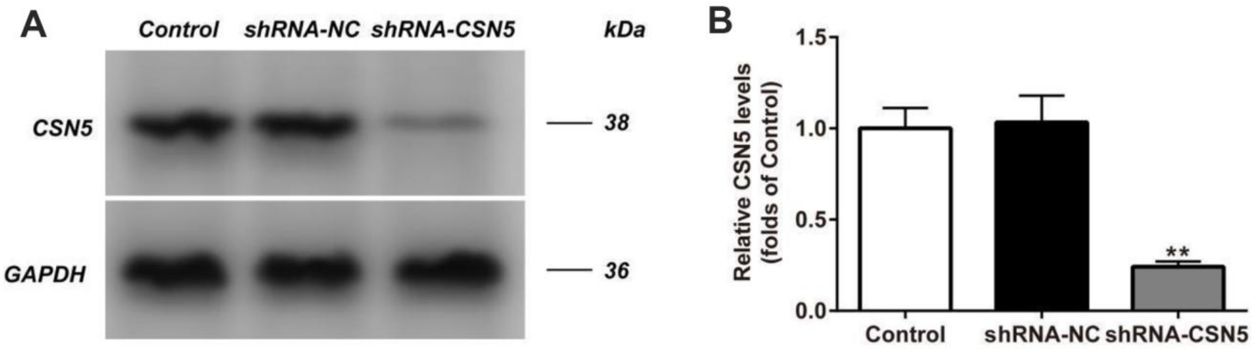

Supplementary Figure 9. The level of CSN5 in GT1-7 was detected by western blot assay and representative bands were shown in (**A**). The level of CSN5 (**B**) was normalized to control. The results were presented as mean ± SD (n = 3). **p < 0.01 vs. shRNA-NC group.

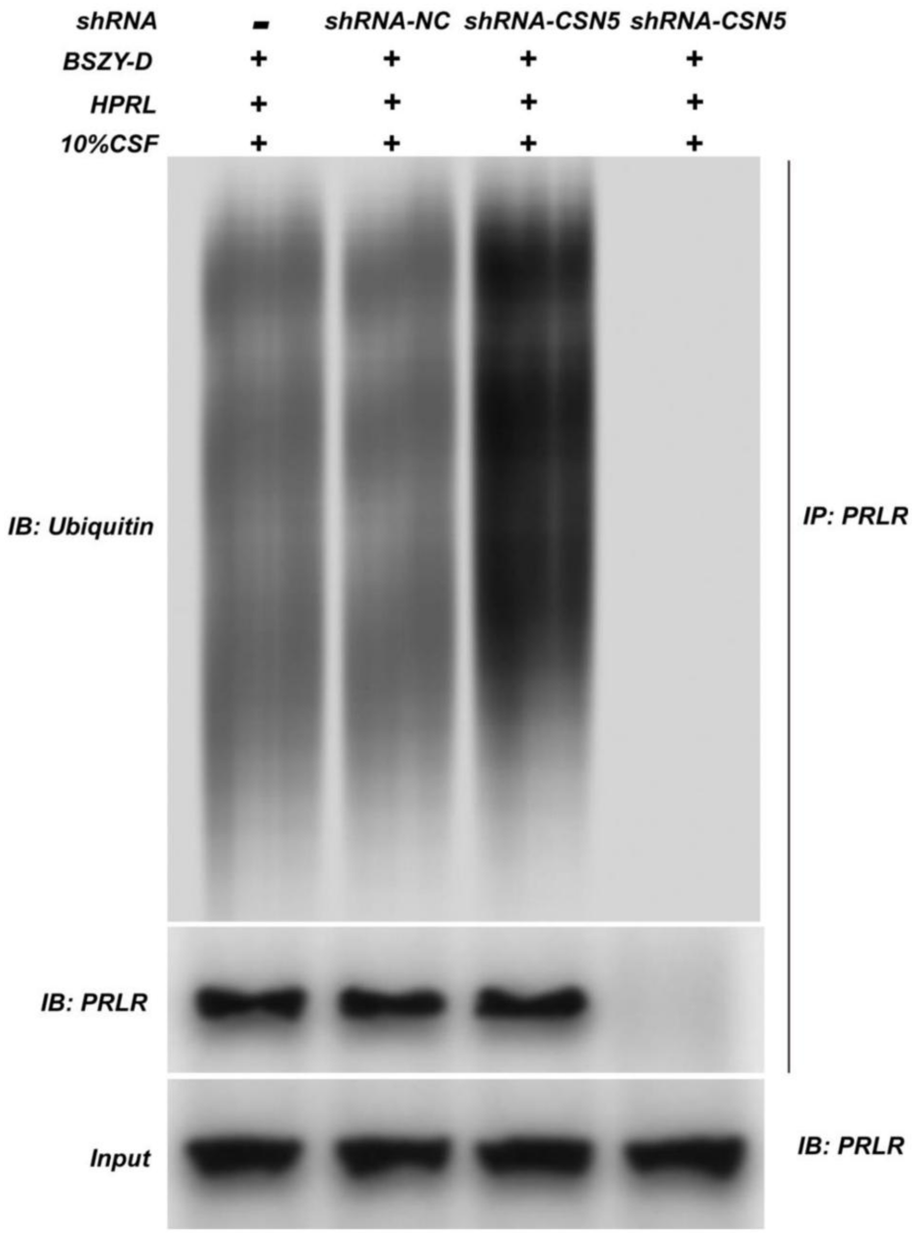

Supplementary Figure 10. The ubiquitin level of PRLR was measured with Co-IP (n = 3).
